# Structure of *Bacteroides thetaiotaomicron* BT2081 at 2.05 Å resolution: the first structural representative of a new protein family that may play a role in carbohydrate metabolism

**DOI:** 10.1107/S1744309110028228

**Published:** 2010-08-04

**Authors:** Andrew P. Yeh, Polat Abdubek, Tamara Astakhova, Herbert L. Axelrod, Constantina Bakolitsa, Xiaohui Cai, Dennis Carlton, Connie Chen, Hsiu-Ju Chiu, Michelle Chiu, Thomas Clayton, Debanu Das, Marc C. Deller, Lian Duan, Kyle Ellrott, Carol L. Farr, Julie Feuerhelm, Joanna C. Grant, Anna Grzechnik, Gye Won Han, Lukasz Jaroszewski, Kevin K. Jin, Heath E. Klock, Mark W. Knuth, Piotr Kozbial, S. Sri Krishna, Abhinav Kumar, Winnie W. Lam, David Marciano, Daniel McMullan, Mitchell D. Miller, Andrew T. Morse, Edward Nigoghossian, Amanda Nopakun, Linda Okach, Christina Puckett, Ron Reyes, Henry J. Tien, Christine B. Trame, Henry van den Bedem, Dana Weekes, Tiffany Wooten, Qingping Xu, Keith O. Hodgson, John Wooley, Marc-André Elsliger, Ashley M. Deacon, Adam Godzik, Scott A. Lesley, Ian A. Wilson

**Affiliations:** a Joint Center for Structural Genomics, http://www.jcsg.org, USA; b Stanford Synchrotron Radiation Lightsource, SLAC National Accelerator Laboratory, Menlo Park, CA, USA; c Protein Sciences Department, Genomics Institute of the Novartis Research Foundation, San Diego, CA, USA; d Center for Research in Biological Systems, University of California, San Diego, La Jolla, CA, USA; e Program on Bioinformatics and Systems Biology, Sanford–Burnham Medical Research Institute, La Jolla, CA, USA; f Department of Molecular Biology, The Scripps Research Institute, La Jolla, CA, USA; g Photon Science, SLAC National Accelerator Laboratory, Menlo Park, CA, USA

**Keywords:** gut microbiome, sugars, structural genomics, immunoglobulin-like fold, jelly-roll fold

## Abstract

BT2081 from *Bacteroides thetaiotaomicron* (GenBank accession code NP_810994.1) is a member of a novel protein family consisting of over 160 members, most of which are found in the different classes of Bacteroidetes. Genome-context analysis lends support to the involvement of this family in carbohydrate metabolism, which plays a key role in *B. thetaiotaomicron* as a predominant bacterial symbiont in the human distal gut microbiome. The crystal structure of BT2081 at 2.05 Å resolution represents the first structure from this new protein family. BT2081 consists of an N-terminal domain, which adopts a β-sandwich immunoglobulin-like fold, and a larger C-terminal domain with a β-sandwich jelly-roll fold. Structural analyses reveal that both domains are similar to those found in various carbohydrate-active enzymes. The C-terminal β-jelly-roll domain contains a potential carbohydrate-binding site that is highly conserved among BT2081 homologs and is situated in the same location as the carbohydrate-binding sites that are found in structurally similar glycoside hydrolases (GHs). However, in BT2081 this site is partially occluded by surrounding loops, which results in a deep solvent-accessible pocket rather than a shallower solvent-exposed cleft.

## Abstract 2

The crystal structure of BT2081 from *B. thetaiotaomicron* reveals a two-domain protein with a putative carbohydrate-binding site in the C-­terminal domain.

## 1. Introduction

*Bacteroides thetaiotaomicron* is a Gram-negative anaerobic bacterium that is a dominant member of the human intestinal tract microbiome. This bacterial species is essential for the metabolism and uptake of dietary plant polysaccharides by the human host (Comstock & Coyne, 2003 ▶; Xu *et al.*, 2003 ▶; Zocco *et al.*, 2007 ▶). To utilize these polysaccharides, *B. thetaiotaomicron* expresses a large number of carbohydrate-processing enzymes. Indeed, it is estimated that ∼6.6% of the *B. thetaiotaomicron* genome encodes such proteins, many of which are novel and have not been functionally or structurally characterized (Davies *et al.*, 2005 ▶).

The BT2081 gene product of *B. thetaiotaomicron* encodes a protein with a molecular weight of 37 198 Da (residues 1–341) and a calculated isoelectric point of 4.9. It contains a predicted N-terminal signalling peptide, suggesting that it is secreted from the cytoplasm. Sequence and genomic context analysis suggest that it is a putative carbohydrate-processing protein that is part of the extracellular polysaccharide-processing machinery. Its N-terminal region (residues 1–114) shares high sequence similarity to the carbohydrate-binding domains of endo-1,4-β-xylanase from *Streptomyces griseoflavus* (35% sequence identity), β-xylosidase from *Magnetospirillum magnetotacticum* (29% sequence identity) and a putative β-fructosidase from *Sarcoptes scabiei* (39% sequence identity), all of which are glycoside hydrolases (GHs), which catalyze the cleavage of the glycosidic bonds in monosaccharide, disaccharides and polysaccharides into their constituent sugar units. Moreover, genome-context analysis shows that BT2081 and several of its paralogs belong to characteristic PULs (polysaccharide-utilization loci) of *B. thetaiotaomicron* and are associated with transmembrane porin domains that are homologous to the *B. thetaiotaomicron* outer membrane protein transporter SusC. SusC is part of the well characterized eight-component *sus* (starch-utilization system) operon used by *B. thetaiotaomicron* in carbohydrate metabolism. Furthermore, in the *sus* operon two other BT2081 paralogs (BT0450 and BT1761) are immediately followed by GHs. To further investigate the role that BT2081 may play in carbohydrate metabolism, we have determined its crystal structure using the semi-automated high-throughput pipeline of the Joint Center for Structural Genomics (JCSG; Lesley *et al.*, 2002 ▶) as part of the Protein Structure Initiative (PSI) of the National Institute of General Medical Sciences, NIH.

## 2. Materials and methods

### 2.1. Protein production and crystallization

Clones were generated using the Polymerase Incomplete Primer Extension (PIPE) cloning method (Klock *et al.*, 2008 ▶). The gene encoding BT2081 (GenBank NP_810994, Swiss-Prot Q8A605) was amplified by polymerase chain reaction (PCR) from *B. thetaiota­omicron* VPI-5482 genomic DNA using *PfuTurbo* DNA polymerase (Stratagene) and I-PIPE (Insert) primers (forward primer, 5′-ctgtacttccagggcCGCGAAGAAGCTCCCAATGCAGAGGCAG-3′; reverse primer, 5′-aattaagtcgcgttaGTCTTCCGAGCGATAGATT­AGTTCGACT-3′; target sequences in upper case) that included sequences for the predicted 5′ and 3′ ends. The expression vector pSpeedET, which encodes an amino-terminal tobacco etch virus (TEV) protease-cleavable expression and purification tag (MGSDK­IHHHHHHENLYFQ/G), was PCR-amplified with V-PIPE (Vector) primers (forward primer, 5′-taacgcgacttaattaactcgtttaaacgg­tctccagc-3′; reverse primer, 5′-gccctggaagtacaggttttcgtgatgatgatgatg­atg-3′). V-PIPE and I-PIPE PCR products were mixed to anneal the amplified DNA fragments together. *Escherichia coli* GeneHogs (Invitrogen) com­petent cells were transformed with the I-PIPE/V-­PIPE mixture and dispensed onto selective LB–agar plates. The cloning junctions were confirmed by DNA sequencing. Using the PIPE method, the gene segment encoding Met1–Ile20 was deleted because it was predicted to be a signal peptide. Expression was performed in selenomethionine-containing medium at 310 K with suppression of normal methionine synthesis. At the end of fermentation, lysozyme was added to the culture to a final concentration of 250 µg ml^−1^ and the cells were harvested and frozen. After one freeze–thaw cycle, the cells were sonicated in lysis buffer [50 m*M* HEPES pH 8.0, 50 m*M* NaCl, 10 m*M* imidazole, 1 m*M* tris(2-carboxyethyl)phosphine–HCl (TCEP)] and the lysate was clarified by centrifugation at 32 500*g* for 30 min. The soluble fraction was passed over nickel-chelating resin (GE Healthcare) pre-equilibrated with lysis buffer, the resin was washed with wash buffer [50 m*M* HEPES pH 8.0, 300 m*M* NaCl, 40 m*M* imidazole, 10%(*v*/*v*) glycerol, 1 m*M* TCEP] and the protein was eluted with elution buffer [20 m*M* HEPES pH 8.0, 300 m*M* imidazole, 10%(*v*/*v*) glycerol, 1 m*M* TCEP]. The eluate was buffer-exchanged with TEV buffer (20 m*M* HEPES pH 8.0, 200 m*M* NaCl, 40 m*M* imidazole, 1 m*M* TCEP) using a PD-10 column (GE Healthcare) and incubated with 1 mg TEV protease per 15 mg eluted protein. The protease-treated eluate was passed over nickel-chelating resin (GE Healthcare) pre-equilibrated with HEPES crystallization buffer (20 m*M* HEPES pH 8.0, 200 m*M* NaCl, 40 m*M* imidazole, 1 m*M* TCEP) and the resin was washed with the same buffer. The flowthrough and wash fractions were combined and concentrated to 13.5 mg ml^−1^ by centrifugal ultrafiltration (Millipore) for crystallization trials. BT2081 was crystallized by mixing 100 nl protein solution with 100 nl crystallization solution in a sitting drop over a 50 µl reservoir volume using the nanodroplet vapor-diffusion method (Santarsiero *et al.*, 2002 ▶) with standard JCSG crystallization protocols (Lesley *et al.*, 2002 ▶). The crystallization reagent consisted of 42%(*v*/*v*) polyethylene glycol 600, 0.25 *M* calcium acetate and 0.1 *M* sodium cacodylate pH 6.33. PEG 400 was added to the crystal as a cryoprotectant to a final concentration of 5%(*v*/*v*). A triangular prism-shaped crystal of approximately 200 × 200 × 200 µm in size was harvested after 43 d at 277 K for data collection. Initial screening for diffraction was carried out using the Stanford Automated Mounting system (SAM; Cohen *et al.*, 2002 ▶) at the Stanford Synchrotron Radiation Lightsource (SSRL, Menlo Park, California, USA). The diffraction data were indexed in the trigonal space group *P*3_2_21. The oligomeric state of BT2081 in solution was determined using a 1 × 30 cm Superdex 200 size-exclusion column (GE Healthcare). The mobile phase consisted of 20 m*M* Tris pH 8.0, 150 m*M* NaCl and 0.02%(*w*/*v*) sodium azide. The molecular weight was calculated using *ASTRA* v.5.1.5 software (Wyatt Technology). Protein concentrations were determined using the Coomassie Plus assay (Pierce).

### 2.2. Data collection, structure solution and refinement

Single-wavelength anomalous diffraction (SAD) data were collected on beamline 11-1 at the SSRL at a wavelength corresponding to the peak (λ_1_) of a selenium SAD experiment. The data set was collected at 100 K using a MAR Mosaic 325 mm CCD detector (Rayonix) using the *Blu-Ice* data-collection environment (McPhillips *et al.*, 2002 ▶). The SAD data were integrated and reduced using *MOSFLM* (Leslie, 1992 ▶) and then scaled with the program *SCALA* (Collaborative Computational Project, Number 4, 1994 ▶). Phasing was performed with *SHELXD* (Sheldrick, 2008 ▶) and *autoSHARP* [mean figure-of-merit (acentric/centric) of 0.28/0.09 with 11 anomalous scatterers per asymmetric unit (eight SeMet, two cacodylate ions and one calcium ion); Vonrhein *et al.*, 2007 ▶]. Automatic model building was performed with *ARP*/*wARP* (Cohen *et al.*, 2004 ▶). Model completion was performed with *Coot* (Emsley & Cowtan, 2004 ▶) and TLS refinement with *REFMAC*5 (Winn *et al.*, 2003 ▶). The refinement included experimental phase restraints in the form of Hendrickson–Lattman coefficients and two TLS groups per chain, with the TLS groups being assigned with the aid of the *TLSMD* server (Painter & Merritt, 2006 ▶). Data-collection and refinement statistics are summarized in Table 1 ▶. X-ray fluorescence emission peaks for selenium, arsenic, nickel and calcium were observed when the crystal was scanned on SSRL beamline 1-5 with X-rays above (500 eV) the Se *K* edge. Calcium was assigned based on binding geometry and its presence in the crystallization conditions. Additional diffraction data were collected above and below the arsenic and nickel edges, with the resultant anomalous electron-density maps revealing the presence of arsenic in the form of cacodylate (which was present in the crystallization condition) and the absence of nickel.

### 2.3. Validation and deposition

**Figure 1:**
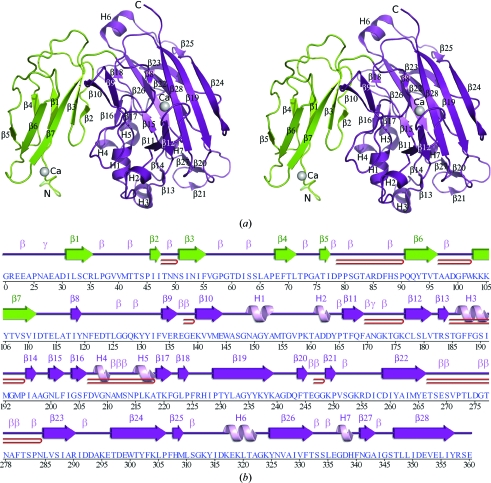
Crystal structure of BT2081 from *B. thetaiotaomicron*. (*a*) Ribbon diagram of the BT2081 monomer, showing the distinct N-terminal (green) and C-terminal (purple) domains. Helices (H1–H7) and β-strands (β1–β28) are indicated. (*b*) Diagram showing the secondary-structure elements of BT2081 superimposed on its primary sequence. The labeling of secondary-structure elements [colored by domain as in (*a*)] is in accord with *PDBsum* (http://www.ebi.ac.uk/pdbsum), in which α-helices (H1, H3, H5 and H6), 3_10_-­helices (H2, H4 and H7) and β-strands (β1–β28) are labeled sequentially, β-turns and γ-turns are designated by Greek letters (β, γ) and β-hairpins by red loops.

The quality of the crystal structure was analyzed using the *JCSG Quality Control* server (http://smb.slac.stanford.edu/jcsg/QC). This server processes the coordinates and data through a variety of validation tools including *AutoDepInputTool* (Yang *et al.*, 2004 ▶), *MolProbity* (Chen *et al.*, 2010 ▶), *WHAT IF* v.5.0 (Vriend, 1990 ▶), *RESOLVE* (Terwilliger, 2004 ▶), *MOLEMAN*2 (Kleywegt, 2000 ▶) as well as several in-house scripts and summarizes the results. Protein quaternary-structure analysis used the *PISA* server (Krissinel & Henrick, 2007 ▶). Fig. 1 ▶(*b*) was adapted from *PDBsum* (Laskowski, 2009 ▶) and all other figures were prepared with *PyMOL* (DeLano Scientific). Atomic coordinates and experimental structure factors for BT2081 from *B. thetaiotaomicron* at 2.05 Å resolution were deposited in the PDB (http://www.pdb.org) under code 3hbz.

## 3. Results and discussion

### 3.1. Overall structure

The crystal structure of BT2081 (Fig. 1 ▶) was determined to 2.05 Å resolution using the SAD method. Data-collection, model and refinement statistics are summarized in Table 1 ▶. The final model includes one BT2081 molecule (residues 21–360), two calcium ions, two sodium ions, two cacodylate anions, two acetate anions, 19 polyethylene glycol molecules and 251 water molecules in the asymmetric unit. Gly0, which is part of the expression construct and remained after cleavage of the N-terminal purification tag, is also part of the final model. The nucleotide sequence corresponding to residues 1–20 was omitted from the expression construct as this region was predicted to encode either a lipoprotein signal peptide or, more likely, a single transmembrane helix anchoring BT2081 in the outer membrane of the cell. Electron density was not observed for the C-­terminal Asp361 or for some of the side-chain atoms of Arg21, Glu23, Glu139, Lys239, Lys249, Glu299, Lys317 and Glu318. The Matthews coefficient (*V*_M_; Matthews, 1968 ▶) for BT2081 is 3.69 Å^3^ Da^−1^ and the estimated solvent content is 67%. The Ramachandran plot produced by *MolProbity* (Chen *et al.*, 2010 ▶) shows that 97.9% and 99.7% of the residues are in the favored and allowed regions, respectively. The single residue in the disallowed region, Ala27, is in a section of poorly defined electron density.

BT2081 is composed of 28 β-strands (β1–β28), four α-helices (H1, H3, H5 and H6) and three 3_10_-helices (H2, H4 and H7) (Fig. 1 ▶). The total β-sheet, α-helical and 3_10_-helical content is 39.0, 6.2 and 2.6%, respectively. Crystallographic packing, as well as *PISA* analyses (Krissinel & Henrick, 2007 ▶) of BT2081, suggest that a monomer is likely to be the biologically relevant form of the protein, which is consistent with results from analytical size-exclusion chromatography (anSEC).

The BT2081 monomer consists of two structural domains (Fig. 1 ▶). The N-terminal domain (residues 21–114) adopts a β-sandwich fold consisting of two-stranded (β1 and β4) and five-stranded (β2, β3 and β5–β7) β-sheets, which concurs with its classification into the immunoglobulin (Ig)-like fold superfamily of SCOP (Andreeva *et al.*, 2004 ▶). The C-terminal domain (residues 115–361) also adopts a β-­sandwich fold, the core of which comprises two five-stranded antiparallel β-sheets that form a concave (β15/β18, β22, β23, β25 and β26) and a convex (β8/β12, β11, β19, β24 and β28) surface. This domain adopts a β-jelly-roll topology, consistent with its assignment to the galactose-binding domain-like superfamily of SCOP.

### 3.2. Similarity to other proteins

Carbohydrate-active enzymes are quite modular, often containing various carbohydrate-binding and catalytic domains in different combinations. Glycoside hydrolases (GHs), for example, often con­tain both catalytic and noncatalytic carbohydrate-binding modules (CBMs) which can be assembled in different orders (Davies *et al.*, 2005 ▶; Henrissat & Davies, 1997 ▶; Cantarel *et al.*, 2009 ▶). CBMs, which can bind a range of different polysaccharides, function to increase the catalytic efficiency of GHs by bringing the catalytic module into closer proximity with its substrate (Bolam *et al.*, 1998 ▶; Tomme *et al.*, 1995 ▶).

**Figure 2:**
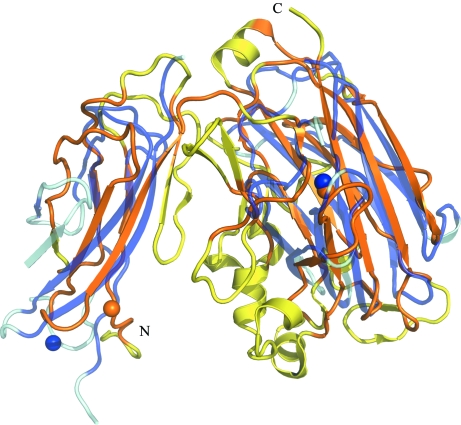
Structural comparison of BT2081 with PKD-CBM44 of *C. thermocellum Ct*Cel9D-Cel44A. Superposition of BT2081 (orange/yellow) with PKD-CBM44 (lilac/pale blue; PDB code 2c26), which also contain both the Ig-like and β-jelly-roll domains. Calcium ions are represented as spheres. Protein regions which were used for alignment by *FATCAT* are shown in darker shades (orange for BT2081 and lilac for PKD-CBM44) to highlight similarities in the protein cores. Please note that two twists have been introduced by *FATCAT* into the structure of PKD-CBM44 in order to obtain optimal full-length alignments of the structures.

The Ig-like and jelly-roll domains of BT2081 have previously been observed as modules in carbohydrate-active enzymes. *Ct*Cel9D-Cel44A, a multi-enzyme GH complex from *Clostridium thermocellum*, for example, contains polycystic kidney disease (PKD) and CBM family 44 (CBM44) domains (PDB code 2c26; Najmudin *et al.*, 2006 ▶) that also adopt Ig-like and β-jelly-roll folds, respectively. However, the relative orientation of the two domains differs significantly and as a result the superposition of these two structures using *FATCAT* (http://fatcat.burnham.org; Ye & Godzik, 2004 ▶) required two rotations or twists around the linker region that connects the two domains in order to obtain optimal alignment of the full-length structures. When BT2081 and PKD-CBM44 are structurally aligned in this way, the r.m.s.d. between 202 C^α^ atoms is 3.15 Å despite only 6.7% sequence identity (Fig. 2 ▶). An important distinction between these two proteins, which highlights the modularity of carbohydrate-active enzymes, is that, in addition to the PKD and CBM44 domains, *Ct*Cel9D-Cel44A contains additional domains (Ig-like, CBM30, GH9 and GH44) that are absent from BT2081.

Similarly, some bacterial sialidases also contain both Ig-like and β-­jelly-roll domains. NedA, a sialidase from *Micromonospora viridi­faciens* (PDB code 1euu; Gaskell *et al.*, 1995 ▶), contains these two domains, but also has a third GH family 33 (GH33) catalytic domain at its N-terminus that adopts a six-bladed β-propeller fold. The C-­terminal β-jelly-roll domain of NedA belongs to CBM family 32 (CBM32), while the middle Ig-like domain is thought to act as a linker region between the CBM32 and GH33 domains. Similar to *Ct*Cel9D-Cel44A, the relative orientation of the Ig-like and β-jelly-roll domains differs significantly between BT2081 and NedA and, as a result, four consecutive twists in the region between these domains were required to obtain an optimal full-length alignment of the structures by *FATCAT*. The resultant r.m.s.d. between 184 C^α^ atoms, which share only 4.8% sequence identity, is 3.11 Å.

### 3.3. BT2081 N-terminal domain

A structural similarity search of the N-terminal domain of BT2081 only performed using the *FATCAT* server, revealed many proteins that belong to the Ig-like fold family. The closest match was to the soluble upper-middle domain (UMD; residues 232–320) of the outer membrane protein usher from *Yersinia pestis* (PDB code 3fcg; Yu *et al.*, 2009 ▶), with an r.m.s.d. of 2.65 Å between 49 C^α^ atoms and a sequence identity of 7.6%. The second and third closest structural neighbors were SoxY from *Paracoccus pantotrophus* (PDB code 2oxg; Sauve *et al.*, 2007 ▶), with an r.m.s.d. of 3.08 Å between 64 C^α^ atoms and a sequence identity of 5.6%, and the I-set domain (residues 3537–3630) of human obscurin (PDB code 2edw; R. Sano, F. Hayashi, M. Yoshida & S. Yokoyama, unpublished work), with an r.m.s.d. of 3.07 Å between 59 C^α^ atoms and a sequence identity of 2.5%. Although none of the top structural neighbors from this search matched carbohydrate-active enzymes, the Ig-like fold has been observed in a number of these proteins. In addition to *Ct*Cel9D-Cel44A and NedA, structures from CBM families 9, 20, 25, 26, 31, 33 and 34 have also been found to adopt Ig-like folds (Hashimoto, 2006 ▶). Despite their structural similarity, the BT2081 N-terminal domain shares little sequence similarity with these CBMs (see Table 2 ▶ *a* for superposition statistics).

Sequence analysis of the BT2081 N-terminal domain with *BLAST* revealed significant sequence similarity to portions of several GHs, including endo-1,4-β-xylanase from *St. griseoflavus* (35% sequence identity over 90 residues), β-xylosidase from *M. magnetotacticum* (29% sequence identity over 94 residues) and a putative β-fructo­sidase from *Sa. scabiei* (39% sequence identity over 76 residues). In all of these sequences, the putative Ig-like domain immediately precedes a putative GH43 catalytic domain, which is known to adopt a fivefold β-propeller fold. The role of the putative Ig-like domain in these GHs is still currently unknown.

**Figure 3:**
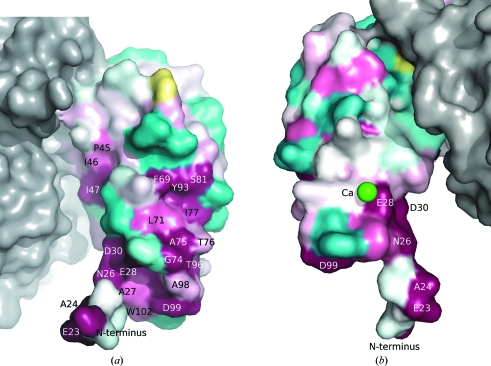
Molecular surface of the BT2081 N-terminal domain colored according to residue conservation by *ConSurf* (http://consurf.tau.ac.il; Landau *et al.*, 2005 ▶). The most conserved residues are shown in magenta, the least conserved residues are shown in cyan and those with insufficient data to determine the conservation level are shown in yellow. The molecular surface of the C-terminal domain is shown in gray. (*a*) and (*b*) are views of opposite surfaces of the N-terminal domain, showing that residue conservation is predominantly on one side. The calcium ion in (*b*) is represented as a green sphere.

A calcium ion is present in the N-terminal domain on the surface near the start of the domain (Fig. 3 ▶). This calcium is octahedrally coordinated by two waters, a cacodylate ion, the carbonyl O atom of Lys103, the carboxylate group of Glu28 and the carboxylate group of Asp162 from a crystallographically related molecule. The role of this calcium (if any) is unclear. CBM9 from *Thermotoga maritima* xylan­ase 10A (PDB code 1i82; Notenboom *et al.*, 2001 ▶) and the PKD domain of *Ct*Cel9D-Cel44A (PDB code 2c26; Najmudin *et al.*, 2006 ▶) also contain calcium(s); however, the location of the calcium-binding site is different from that in BT2081. It has been postulated that these calcium ions may play a structural role given the nature of the buried binding sites (Najmudin *et al.*, 2006 ▶; Notenboom *et al.*, 2001 ▶). Unlike those proteins, however, the calcium in the BT2081 N-terminal domain is quite solvent-exposed and, as three of the six ligands are solvent molecules and another is a crystallographically related molecule, this bound calcium ion is most likely to be an artifact of crystallization.

Analysis of BT2081 and its homologs reveals a highly conserved region of the N-terminal domain that may be functionally important. These conserved residues include Glu23, Ala24, Asn26, Glu28 and Ile31 situated between the N-terminus and β-strand 1, as well as residues from two adjacent regions: Phe69, Gly74, Ala75 and Ile77 between β-strands 4 and 5, and Val95, Thr96, Asp99 and Trp102 between β-strands 6 and 7. Together, these residues form a highly conserved patch on one surface of the N-terminal domain, in contrast to the minimal conservation that is observed on the opposite surface (Fig. 3 ▶).

In light of the fact that Ig-like domains in GHs are sometimes CBMs, one possibility is that the N-terminal domain of BT2081 may also be a CBM and, as such, the highly conserved region may be a carbohydrate-binding site. Another possible role for the BT2081 N-­terminal domain is as a linker region, similar to the Ig-like domain in NedA. BT2081 is predicted to contain a signal sequence at the N-­terminus, which, based on its predominantly hydrophobic amino-acid composition, may correspond to a single transmembrane helix that anchors BT2081 to the bacterial outer membrane. If this is indeed the case, the N-terminal domain may act as a linker region between the membrane surface and the C-terminal domain.

### 3.4. BT2081 C-terminal domain

Analysis with *FATCAT* indicates that most of the top structural neighbors of the BT2081 C-terminal domain are either catalytic modules or CBMs of GHs. Representative examples of these structural neighbors are the catalytic module of a *Bacillus* 1,3–1,4-β-glucanase (PDB code 1byh; Keitel *et al.*, 1993 ▶) and the CBMs from CBM families 6, 11, 16 and 29, which include *C. thermocellum* xylan­ase 11A (PDB code 1gmm; Czjzek *et al.*, 2001 ▶), *C. thermocellum* Lic26A-Cel5E endoglucanase (PDB code 1v0a; Carvalho *et al.*, 2004 ▶), *Thermoanaerobacterium polysaccharolyticum* mannanase (PDB code 2zew; Bae *et al.*, 2008 ▶) and *Piromyces equi* cellulose/hemicellulase complex (PDB code 1gwk; Charnock *et al.*, 2002 ▶), respectively (see Table 2 ▶ *b*).

**Figure 4:**
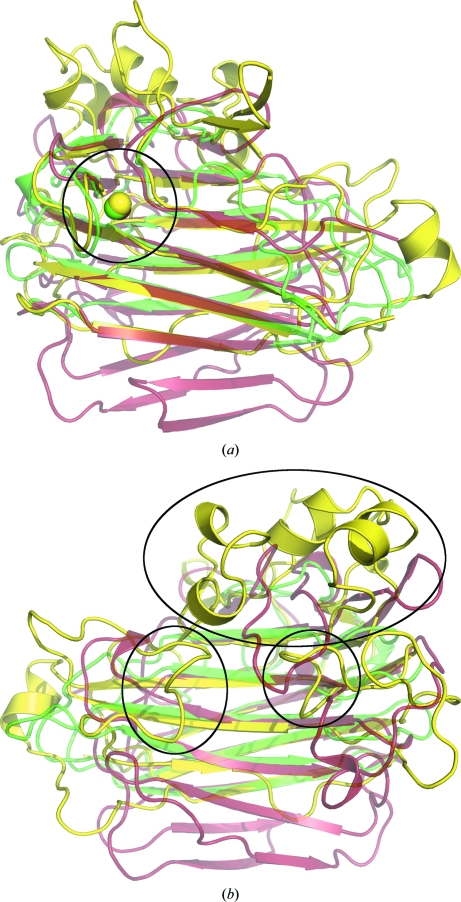
Superposition of the BT2081 C-terminal domain (yellow) with two representative top structural neighbors as assessed by *FATCAT*: the catalytic module of a *Bacillus* 1,3–1,4-β-glucanase (red; PDB code 1byh) and CBM29 of a mannanase from *T. polysaccharolyticum* (green; PDB code 2zew). (*a*) View from above the convex surface of the β-jelly-roll core, highlighting the conserved calcium ion represented as spheres and circled. (*b*) View from above the concave surface of the β-jelly-roll core, highlighting the extended loop regions of BT2081 (circled) which help to form a pocket on the concave surface of the core.

**Figure 5:**
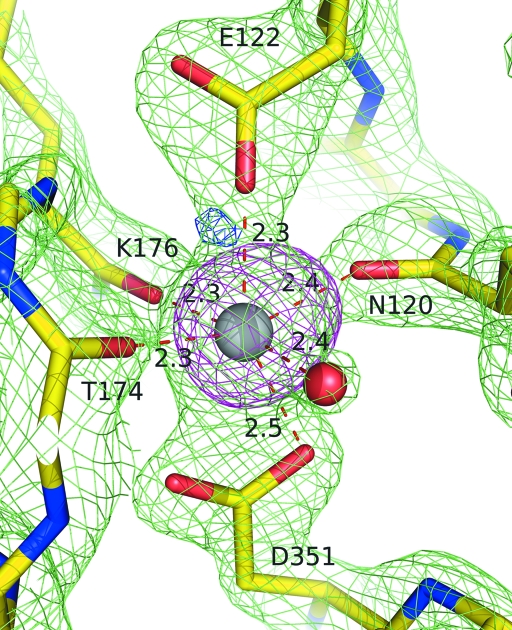
Coordination of the conserved C-terminal calcium ion of BT2081. Electron density from 2*F*_o_ − *F*_c_ (contoured at 2.5σ level) and *F*_o_ − *F*_c_ (contoured at 3.0σ level) maps is represented as green and blue mesh, respectively. A 13σ level anomalous signal obtained from data collected below the nickel edge was seen for the calcium and is shown here contoured at the 6.0σ level as magenta mesh. Distances between the calcium ion and its ligands are indicated in Å.

Despite the low sequence identity between the C-terminal domain of BT2081 and its top structural neighbors, they share the same core β-jelly-roll topology (Fig. 4 ▶). Another shared feature is a conserved calcium-binding site on the convex surface of the β-jelly roll (Fig. 5 ▶). The role of this conserved calcium is most likely to be structural, as it has previously been shown in the case of CBM4 to contribute greater stability to the protein fold (Abou-Hachem *et al.*, 2002 ▶). In BT2081, this calcium is octahedrally coordinated by a water molecule, the carbonyl O atoms of Asn120, Thr174 and Lys176 and the carboxylate O atoms of Glu122 and Asp351. Of this cluster, Glu122 and Asp351 are strictly conserved, Lys176 is highly conserved and Asn120 and Thr174 are poorly conserved among BT2081 sequence homologs, although the latter three would not necessarily be expected to be conserved in amino-acid type as they only make main-chain contacts to the calcium. Several of the immediate neighboring residues (*e.g.* Phe133, Trp145, Lys237, Trp300 and Phe331) are also highly con­served. Comparison of this site in BT2081 and its structural neighbors reveals that it is most similar, in terms of residue conservation, to the corresponding sites in members from the CBM11 (PDB code 1v0a) and CBM16 (PDB code 2zew) families.

### 3.5. Putative carbohydrate-binding pocket in the C-terminal domain

The C-terminal domain of BT2081 contains extended solvent-exposed loop regions (Glu144–Pro165, Thr182–Gly199, Ile203–Ile228, Ala240–Cys258, Thr267–Asn284, Phe307–Lys324 and Ser333–Ser346) that are not found in its closest structural neighbors. Several of these loops also contain additional secondary-structural elements including β-strands, α-helices and 3_10_-helices. As a consequence of these extended loop regions, a significant distinction between BT2081 and its structural counterparts is the presence of a deep solvent-accessible pocket in BT2081 formed by highly conserved residues from the concave surface of the β-jelly-roll core and five of the loop regions extending from this core. The concave surface of the β-jelly-roll core forms the base of this pocket, with loop 1 (residues 144–165) forming the pocket terminus, loop 2 (residues 182–199) and loop 3 (residues 203–228) comprising the middle section of the pocket, and loop 4 (residues 267–284) and loop 5 (residues 333–346) forming the side walls near the pocket entrance.

**Figure 6:**
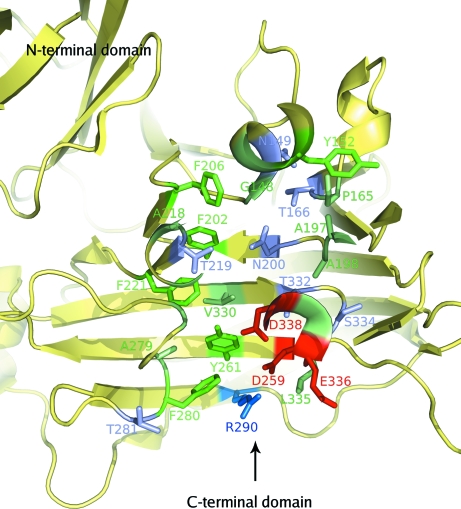
Putative carbohydrate-binding pocket as seen from above the concave surface of the jelly-roll fold. An arrow indicates the pocket entrance. The residues that line the pocket are highlighted in stick representation and are color-coded according to type as follows: aromatic, green; hydrophobic, light green; polar, lilac; acidic, red; basic, blue.

The pocket is lined with a combination of both hydrophobic and hydrophilic residues (aliphatic, Gly148, Gly151, Pro165, Gly189, Met194, Ile196, Ala197, Ala198, Ala211, Met212, Pro215, Leu216, Ala218, Gly276, Ala279, Val330, Leu335 and Gly337; aromatic, Tyr152, Phe202, Phe206, Phe221, Tyr261, Phe280 and Phe340; hydrophilic, Asn149, Thr155, Thr166, Thr185, Asn200, Thr219, Asp259, Thr281, Arg290, Thr332, Ser334, Glu336, Asp338 and His339; Fig. 6 ▶), most of which are highly conserved among BT2081 and its top 20 *PSI-BLAST*-derived sequence homologs, lending support to this pocket being functionally significant.

**Figure 7:**
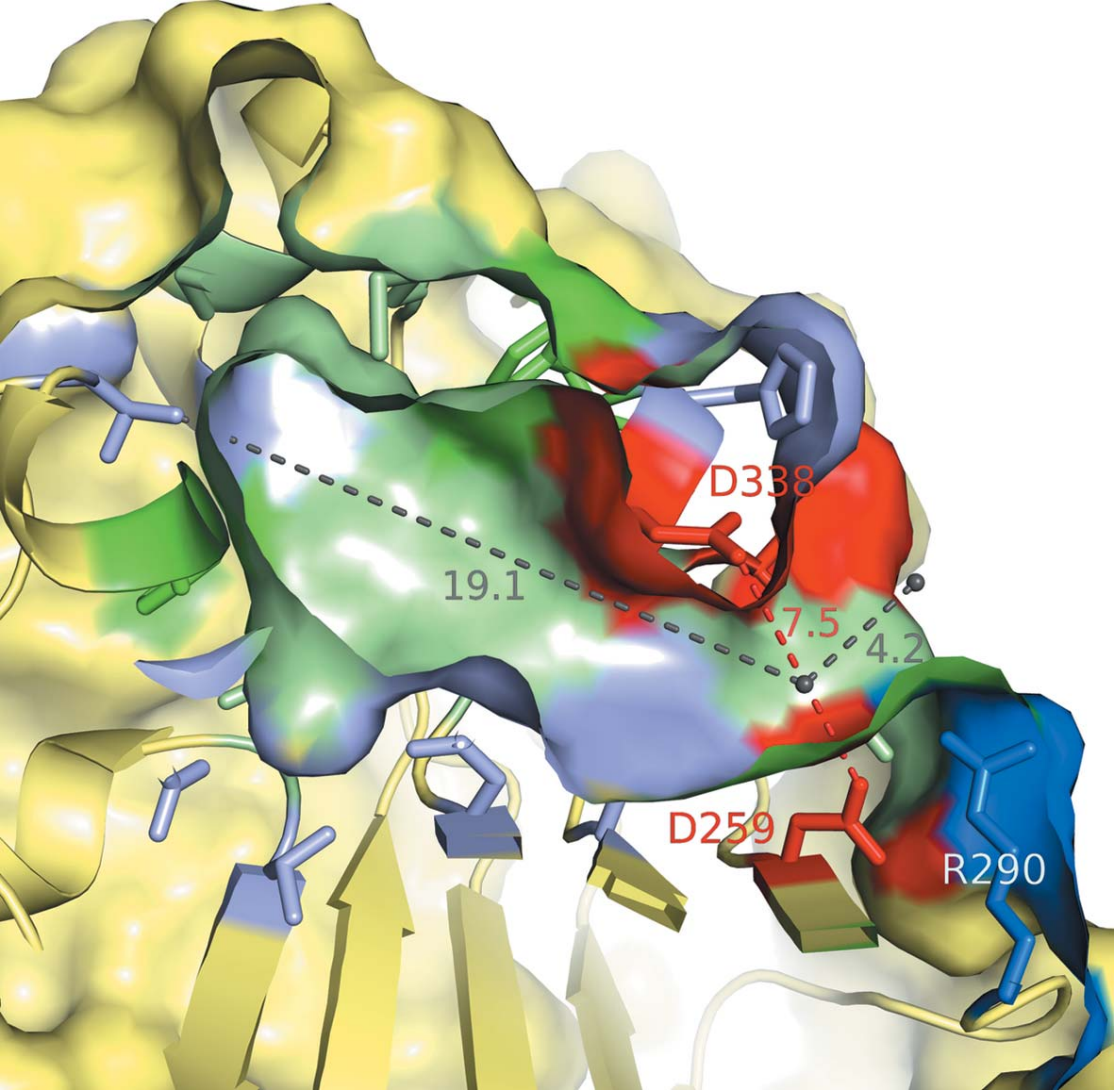
Cutaway side view of the putative carbohydrate-binding pocket highlighting the nature of the residues lining the pocket, including several that may play a role in catalysis. The color scheme of the residues is the same as in Fig. 6 ▶.

The pocket, which has a volume of ∼1160 Å^3^ as calculated by *PDBsum* (Laskowski, 2009 ▶), has a slight kink near its entrance and measures ∼4 Å from the entrance to this bend and ∼19 Å from there to the bottom of the pocket. The width varies along the length of the pocket, ranging from ∼10 Å at the entrance to ∼7.5 Å at the narrowest portion at the bend and widening to ∼13.5 Å near the pocket terminus (Fig. 7 ▶).

Several features of this pocket suggest that it may be a carbo­hydrate-binding site. First, it is located in a similar location to the carbohydrate-binding sites of structurally similar GH catalytic modules and CBMs. In CBMs that can be classified as ‘glycan-chain-binding’ or type B CBMs (Boraston *et al.*, 2004 ▶), the carbohydrate-binding site is a cleft that extends along the concave surface of the β-­jelly-roll fold. These clefts consist of several subsites to which individual sugar moieties of a polysaccharide chain can bind. The topography of the cleft is defined in a large part by several aromatic residues, which are key determinants of polysaccharide binding specificity. Second, hydrogen bonding between the polysaccharide and polar residues in the binding site is also important for binding affinity in these CBMs (Boraston *et al.*, 2004 ▶).

Similar to the carbohydrate-binding sites found in type B CBMs, the putative binding pocket of BT2081 contains several highly con­served aromatic residues arranged in such a way that they form a twisted platform to which a potential polysaccharide could bind. These aromatic residues include Phe202, Phe206, Phe221, Tyr261, Phe280 and Phe340, of which Phe202, Phe206, Tyr261 and Phe340 are strictly conserved among sequence homologs. Moreover, the BT2081 pocket contains a number of polar residues (*e.g.* Asn149, Thr155, Thr166, Thr185, Asn200, Thr219 and Thr332) whose side chains are positioned for potential hydrogen bonding with a bound polysaccharide, in another characteristic feature which helps to define carbohydrate-binding specificity in type B CBMs.

The evidence from genomic context analysis and the similarity of the fold and putative binding pocket of BT2081 to those of CBMs and catalytic modules of GHs strongly suggest that the pocket in BT2081 may be a carbohydrate-binding site. If so, the next question is whether the C-terminal domain of BT2081 displays GH enzymatic activity or whether it acts instead as a noncatalytic CBM.

### 3.6. BT2081 as a potential GH

The hydrolysis of glycosidic bonds by GHs usually occurs *via* general acid catalysis involving two GH carboxylate residues that act as a proton donor and a nucleophile/base (Koshland, 1953 ▶; Sinnott, 1990 ▶). Hydrolysis can occur *via* two canonical mechanisms, which result in either the inversion or retention of the configuration of the anomeric carbon that undergoes nucleophilic attack.

In *Bacillus* 1,3–1,4-β-glucanase (PDB code 1byh), a member of GH family 16 (GHF16) and one of the top structural neighbors of the BT2081 C-terminal domain, Glu105 and Glu109 act as the nucleophile and acid/base in a retaining mechanism for the glycosidic bond cleavage (Keitel *et al.*, 1993 ▶). A comparison with the BT2081 pocket reveals that BT2081 lacks these two catalytic glutamate residues at the corresponding locations. However, several other charged residues line the pocket, including Asp259, Arg290, Glu336 and Asp338, all of which are near each another at the pocket entrance (Figs. 5 ▶, 6 ▶ and 7 ▶). Of these residues, Asp259 and Asp338 at the bend of the pocket are particularly interesting candidates for performing catalytic roles because they are positioned opposite each other in the pocket, with their carboxylate O atoms being sufficiently far apart (∼7.5 Å) to accommodate a carbohydrate substrate. The feasibility of Asp259 as a potential catalytic residue in a hydrolysis reaction may further be supported by its interaction with the neighboring Arg290, which would be expected to reduce the p*K*_a_ of Asp259 and thereby prime it for a role as a nucleophile in a retaining mechanism (Fig. 7 ▶).

**Figure 8:**
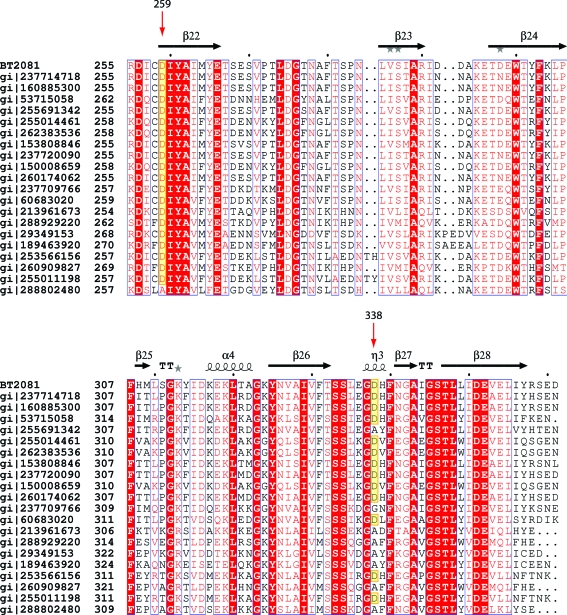
Sequence alignment between BT2081 and its top 20 *PSI-BLAST* homologs (the alignment  is only shown for residues 255–361 of BT2081 for clarity). Residues in white characters on a red background are strictly conserved, while those in red characters with a white background are highly conserved. Red arrows indicate the two residues of BT2081 (Asp259 and Asp338) which may play a role in catalysis. Sequences with aspartates at these positions are highlighted in yellow.

Among the top 20 BT2081 homologs derived from *PSI-BLAST* Asp259 is almost absolutely conserved (19/20 = 95%), while Asp338 (12/20 = 60%) is less so (Fig. 8 ▶). In the eight sequences in which the position equivalent to Asp338 is not an aspartate, seven have an alanine and one has a glycine. The lack of strict conservation at these sites, particularly Asp338, may raise doubts as to the functional role of these residues; however, such an apparent lack of conservation among active-site residues is not unprecedented. In GH family 97 (GH97), for example, its members have evolutionarily diverged into two main subfamilies which differ in their catalytic mechanism, so that the members of one subfamily are retaining GHs while the members of the other are inverting GHs. In addition to these two main subfamilies, six outlier sequences in the GH97 family were found which lacked key catalytic residues, indicating that these members may be inactive or have evolved a different catalytic mechanism (Gloster *et al.*, 2008 ▶). It is feasible that a similar evolutionary divergence may also have occurred in the BT2081 protein family.

The fact that the putative carbohydrate-binding site in BT2081 is a pocket rather than a tunnel or a cleft, as seen in other carbohydrate-active enzymes, suggests that BT2081, if it were indeed a GH, would be likely to be classified as an ‘exo’ GH (*i.e.* it would cleave the polysaccharide chain at its ends rather than in the middle) and in this regard would be similar to other exopolysaccharidases, such as glucoamylase and β-amylase (Davies & Henrissat, 1995 ▶).

Manual docking of various polysaccharides (*e.g.* cellopentaose, mannopentaose and cellohexaose) into the BT2081 pocket reveals that the pocket can accommodate a polysaccharide as large as a pentaose, with three of the sugar units fitting into the section of the pocket extending from the bend to the pocket terminus and the other two sugar units fitting into the portion that extends from the bend to the pocket entrance. Consequently, if BT2081 were indeed proven to be a GH with Asp259 and Asp338 being the catalytic residues, it would be feasible that up to three terminal sugar units of a bound polysaccharide chain could be cleaved off at one time.

## 4. Conclusions

BT2081 is the first structural representative of a new protein family which is likely to play a role in carbohydrate metabolism in the distal human gut based on structural and genomic context analyses. BT2081 contains Ig-like and jelly-roll domains, which have been observed to be modules in carbohydrate-active enzymes, such as GHs. The N-­terminal Ig-like domain may act as a CBM and/or a linker region between the outer membrane and the C-terminal domain. The C-­terminal domain contains a pocket that is similar in many respects to carbohydrate-binding sites in CBMs and catalytic modules of GHs. This domain may be catalytic, with Asp259 and Asp338 acting as the catalytic residues in glycosidic bond cleavage. Further biochemical investigation of BT2081 is needed to ascertain whether this is indeed the case.

Additional information about BT2081 is available from *TOPSAN* (Krishna *et al.*, 2010 ▶) at http://www.topsan.org/explore?PDBid=3hbz.

## Supplementary Material

PDB reference: putative glycoside hydrolase, 3hbz

## Figures and Tables

**Table 1:** Summary of crystal parameters, data-collection and refinement statistics for BT2081 (PDB code 3hbz) Values in parentheses are for the highest resolution shell.

Space group	*P*3_2_21
Unit-cell parameters (Å)	*a* = 94.55, *b* = 94.55, *c* = 107.81
Data collection	
Wavelength (Å)	0.9785
Resolution range (Å)	29.8–2.05 (2.10–2.05)
No. of observations	268747
No. of unique reflections	35437
Completeness (%)	99.9 (99.9)
Mean *I*/σ(*I*)	16.7 (1.8)
*R*_merge_ on *I*† (%)	0.091 (0.75)
Model and refinement statistics	
Resolution range (Å)	29.8–2.05
No. of reflections (total)	35400‡
No. of reflections (test)	1773
Completeness (%)	99.9
Cutoff criterion	|*F*| > 0
*R*_cryst_§	0.159
*R*_free_¶	0.191
Stereochemical parameters	
Restraints (r.m.s.d. observed)	
Bond angles (°)	1.60
Bond lengths (Å)	0.018
Average isotropic *B* value (Å^2^)	42.9††
ESU‡‡ based on *R*_free_ (Å)	0.121

† *R*_merge_ = 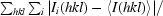 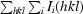.

‡ Typically, the number of unique reflections used in refinement is slightly less than the total number that were integrated and scaled. Reflections are excluded owing to systematic absences, negative intensities and rounding errors in the resolution limits and unit-cell parameters.

§ *R*_cryst_ = 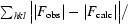 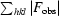, where *F*_calc_ and *F*_obs_ are the calculated and observed structure-factor amplitudes, respectively.

¶ *R*_free_ is the same as *R*_cryst_ but for 5.0% of the total reflections chosen at random and omitted from refinement.

†† This value represents the total *B* that includes TLS and residual *B* components.

‡‡ Estimated overall coordinate error (Collaborative Computational Project, Number 4, 1994 ▶; Cruickshank, 1999 ▶).

**(a):** Superposition of the BT2081 N-terminal domain with CBM families that adopt Ig-like folds.

PDB code	CBM family	Optimized r.m.s.d. (Å)	Equivalent positions (No. of C^α^ atoms)	Sequence identity (%)	*P*-value†
1i82	CBM9	3.83	67	3.3	2.94 × 10^−1^
1b90	CBM20	3.39	43	9.1	8.34 × 10^−1^
2c3v	CBM25	3.01	50	6.5	1.29 × 10^−1^
2c3g	CBM26	3.45	56	3.6	2.05 × 10^−1^
2cov	CBM31	3.43	65	5.5	7.31 × 10^−2^
2bem	CBM33	3.09	72	4.7	2.71 × 10^−1^
1bvz	CBM34	3.14	59	2.6	7.30 × 10^−1^

**(b):** Representative closest structural neighbors of the BT2081 C-terminal domain.

PDB code	CBM/GH family	Optimized r.m.s.d. (Å)	Equivalent positions (No. of C^α^ atoms)	Sequence identity (%)	*P*-value†
1gwk	CBM29	3.08	128	6.8	3.22 × 10^−4^
2zew	CBM16	3.01	136	10.4	9.93 × 10^−4^
1v0a	CBM11	3.05	130	9.8	1.78 × 10^−3^
1byh	GH16	3.04	137	5.0	1.79 × 10^−3^
1gmm	CBM6	3.05	115	4.6	1.95 × 10^−3^

† The *FATCAT* *P*-value measures the probability of obtaining a similar result between two random structures. This *P*-value is calculated based on empirical fitting of the extreme value distribution to the *FATCAT* similarity score (Ye & Godzik, 2004 ▶). The smaller the *P*-value, the more statistically significant the similarity between corresponding structures (*P*-values of <0.05 are considered to be significant).
